# 16S rDNA-based detection technology: use in lambs infected with *Nematodirus oiratianus* to analyze changes in intestinal flora

**DOI:** 10.1051/parasite/2025052

**Published:** 2025-09-15

**Authors:** Si-Yao Li, Bin Hou, Qiqige Wuyun, Zhula Eerdeni, Dare Zang, Surong Hasi

**Affiliations:** 1 Key Laboratory of Clinical Diagnosis and Treatment Technology in Animal Diseases, Ministry of Agriculture, College of Veterinary Medicine, Inner Mongolia Agricultural University Hohhot 010000 PR China; 2 Wushen Animal Disease Prevention and Control Center Ordos 017000 PR China; 3 Ordos Vocational College of Eco-Environment Ordos 017010 PR China

**Keywords:** Lamb, *Nematodirus oiratianus*, Intestinal flora, 16S rDNA, Bioinformatics analysis

## Abstract

The nematode *Nematodirus oiratianus* is associated with major economic losses in the livestock industry, as it is a common gastrointestinal parasites of cattle, sheep, and other ruminants. These parasites primarily obtain nutrients by consuming the blood of their host. This study aimed to investigate changes in the intestinal microbiota of lambs infected with *N. oiratianus* before and after infection, using 16S rDNA sequencing technology. We aimed to reveal the impact of *N. oiratianus* infection on lamb intestinal microecology and to provide scientific evidence for the prevention and control of related diseases. Compared with the infected group, the control group had more bacterial species. Chao, Ace, and Shannon indices were significantly lower in the infected group (*p* < 0.05), while the Simpson index showed no significant difference (*p* > 0.05). These findings collectively indicate significant divergence in the composition of bacterial taxa between the infected and control groups. The phylum with the highest relative abundance in both groups was Firmicutes, followed by Bacteroidetes. Linear Discriminant Analysis Effect Size (LEfSe) identified significantly enriched taxa, including Proteobacteria, *Bacteroides*, and Campylobacteria in the control group, and Clostridiales, Firmicutes, and Ruminococcaceae in the infected group. Functional predictions indicated that the altered microbiota was associated with metabolic pathways such as carbohydrate, amino acid, and vitamin metabolism. Infection with *N. oiratianus* led to significant alterations in the diversity and composition of the intestinal microbiota in lambs.

## Abstract (fr)

Le nématode *Nematodirus oiratianus* est à l’origine de pertes économiques importantes dans l’élevage ; c’est un parasite gastro-intestinal fréquent chez les bovins, les ovins et autres ruminants. Ces parasites se nourrissent principalement du sang de leur hôte. Cette étude visait à analyser les modifications du microbiote intestinal d’agneaux infectés par *N. oiratianus* avant et après l’infection, grâce à la technologie de séquençage de l’ADNr 16S. Notre objectif était de révéler l’impact de l’infection par *N. oiratianus* sur la microécologie intestinale de l’agneau et de fournir des preuves scientifiques pour la prévention et le contrôle des maladies associées. Comparé au groupe infecté, le groupe témoin présentait davantage d’espèces bactériennes. Les indices Chao, Ace et Shannon étaient significativement plus faibles dans le groupe infecté (*p* < 0,05), tandis que l’indice de Simpson ne présentait aucune différence significative (*p* > 0,05). Ces résultats indiquent collectivement une divergence significative dans la composition des taxons bactériens entre les groupes infecté et témoin. L’embranchement présentant l’abondance relative la plus élevée dans les deux groupes était celui des Firmicutes, suivi des Bacteroidetes. L’analyse LEfSe a identifié des taxons significativement enrichis, notamment les Proteobacteria, les *Bacteroides* et les Campylobacteria dans le groupe témoin, et les Clostridiales, les Firmicutes et les Ruminococcaceae dans le groupe infecté. Les prédictions fonctionnelles ont indiqué que le microbiote altéré était associé à des voies métaboliques telles que le métabolisme des glucides, des acides aminés et des vitamines. L’infection par *N. oiratianus* a entraîné des altérations significatives de la diversité et de la composition du microbiote intestinal chez les agneaux.

## Introduction

*Nematodirus oiratianus* is a gastrointestinal nematode that affects livestock. It primarily obtains nutrients by feeding on the host’s blood, which poses a significant threat to livestock [18]. In our country, especially among sheep, the gastrointestinal nematode infection rate and intensity are very high, causing significant economic losses to the livestock industry [2, 12]. In addition, nematode infections caused by this parasite have a global distribution among livestock [20, 27].

In recent years, as the impact of gut microbiota on diseases has become increasingly recognized, research targeting gut microbiota for disease intervention has gradually become a research hotspot. Gut microbiota are a complex microbial community composed of bacteria, fungi, viruses, archaea, and other microorganisms coexisting in the gastrointestinal tract, often referred to as the “hidden organ” within animals. There is a bidirectional interaction between the host and gut microbiota, and this interaction is regulated by the diversity and abundance of the microbiota, which in turn affects the host’s health [6]. The microbial community in the gut not only directly participates in the digestion and absorption of substances within the host intestine, but also plays a role in regulating the immune system, serving as an important site for defending against pathogenic invasions. It is crucial for the growth, development, and health of both humans and animals [28].

In recent years, with the continuous advancement of genomic [4] and next-generation sequencing (NGS) [19] technologies, research on microbial community structures has gradually shifted from traditional pure-culture extraction methods to high-throughput sequencing. 16S rDNA [27] as a novel high-throughput sequencing technology, can more comprehensively reveal the structure and spatial distribution of microbial communities. It is currently widely used for the comparative analysis of differences in gut microbial community structures.

This study aimed to focus on changes in the gut microbiota of lambs during the infection process of *Nematodirus oiratianus*, using a 16S rDNA sequencing method. The research findings will help to deepen our understanding of the impact of nematode infection on the gut microbiome balance of the host and provide new theoretical foundations for the prevention and control of nematode infections. It will also serve as a reference for research on intestinal parasitic diseases and other related intestinal diseases.

## Materials and methods

### Ethics statement

Animal procedures were performed following the National Standard Guideline for Ethical Review of Animal Welfare (GB/T 35892-2018) and approved by the Animal Care and Use Committee of Inner Mongolia Agricultural University.

### Eggs

Eggs were preserved by the College of Veterinary Medicine, Inner Mongolia Agricultural University. They were incubated at 28 °C for 14 days to obtain infectious larvae (L3).

### Experimental animal grouping and treatment

Twelve six-month-old lambs were purchased from the Wushenqi area of Ordos City, Inner Mongolia Autonomous Region. All the lambs were in good health, with 6 rams and 6 ewes. Subsequently, the researchers randomly divided the lambs into an experimental group (*n* = 6) and a control group (*n* = 6). When grouping, it was strictly ensured that the gender composition of the two groups was consistent, that is, each group consisted of 3 rams and 3 ewes. After 7 days of acclimatization, anthelmintic treatment was administered using avermectin (0.3 mg/kg), closantel sodium (5–10 mg/kg), niclosamide (50–75 mg/kg), and sulfaquinoxaline sodium (diluted before use, 300 mg/L). After deworming, the lambs were kept under the same feeding conditions for 14 days. After 14 days, the experimental group was orally administered 10,000 infectious larvae of *Nematodirus oiratianus*. The uninfected group was fed normally under the same conditions until day 60. During this period, fecal samples were collected from the rectum and placed in a liquid nitrogen tank. Immediately after the collection, the fecal samples of the lambs in each group were sent to BGI Shenzhen for 16S rDNA sequencing to analyze the composition and structure of the gut microbiota in the feces.

### 16S rDNA sequencing

#### Sample DNA extraction

100–200 mg of the sample was placed in a centrifuge tube filled with grinding beads; 1 mL of ATL/PVP-10 buffer was added; the sample was ground on a high-speed mill and then incubated at 65 °C for 20 min, then placed in a centrifuge at 14,000 × *g* for 5 min. The supernatant was transferred to a new centrifuge tube, 0.6 mL of PCI buffer was added, and the samples were vortexed and mixed for 15 s. Finally, the supernatant was transferred to a deep-well plate with magnetic bead binding, including 600 μL buffer, 20 μL proteinase K, 5 μL RNase A, 700 μL Wash 1, 700 μL Wash 2, 700 μL Wash 3, and 100 μL elution buffer. The Kingfisher selection program was started, each deep-well plate placed in the corresponding position of the instrument, and the program run. Once the program was complete, the DNA solution was transferred from the elution buffer deep-well plate to a 1.5 mL centrifuge tube for storage.

#### Library preparation and sequencing

The V3-V4 variable region of 16S rDNA was amplified using 2 × Phanta Max Master Mix polymerase and PCR upstream and downstream degenerate primers (338F: 5′-ACTCCTACGGGAGGCAGCAG-3′, 806R: 5′-GGACTACHVGGGTWTCTAAT-3′). The PCR reaction was 50 μL containing 30 ng of template and fusion PCR primers. The PCR procedure was as follows: 95 °C for 3 min; 30 cycles: 95 °C for 15 s, 56 °C for 15 s, 72 °C for 45 s; extension at 72 °C for 5 min at the end. PCR products were purified using DNA sorting magnetic beads, and PE300 sequencing was performed on the Illumina Miseq sequencing platform using QC-checked libraries.

#### Data filtering

The raw sequencing data underwent a series of processing steps to yield high-quality clean data suitable for analysis. Specifically, the following software and their respective versions were employed: for quality control (QC), iTools Fqtools fqcheck (v. 0.25) was utilized. To remove junctions and primers, cutadapt (v. 2.6) was applied. Finally, the readfq software (v. 1.0) was used for the filtering process, ensuring that only reliable data remained for subsequent analysis.

#### Tags connection, OTU clustering

When the Usearch method was used for clustering, the sequence splicing software Fast Length Adjustment of Short reads, v1.2.11, was used to assemble the pairwise reads obtained by double-ended sequencing into a single sequence by using the overlap relation, and the tags of the hypervariable region were obtained. The reuse software USEARCH (v7.0.1090) clusters the spliced tags into OTUs. The main process was as follows: uparse was used to cluster under 97% similarity to obtain representative sequences of OUT; removal of chimerism from PCR amplification from OTU representative sequences using UCHIME (v4.2.40) (chimerism database: gold database (v20110519)); align all tags back to the OTU representative sequence using the usearch_global method to obtain a statistical table of the abundance of OTUs for each sample.

#### OTU species notes

After obtaining the OTU representative sequences, we used RDP classifier software to compare the OTU representative sequences with the database [38] for species annotation, with a confidence threshold set to 0.6. We filtered the annotation results as follows: we removed OTUs with no annotation results; and removed species whose annotation results are not part of the analyzed project. Based on the OTU and annotation results, statistical analysis and visualization of the microbial community composition and structure were performed. By drawing Venn diagrams, the number of common and unique OTUs in multiple samples can be displayed, providing a visual representation of the overlap of OTUs between samples. OTU accumulation curves were plotted to reflect the impact of sampling individuals on species diversity. Rarefaction curves were plotted to determine the appropriate sequencing depth for the samples.

#### Alpha diversity

MOTHUR (v.1.31.2) software was used for α-diversity, which reflects the diversity, community richness, and evenness of species within an ecosystem, a significance level of *α* = 0.05 and *p* < 0.05 was considered statistically significant.

#### Beta diversity

β-diversity analysis was used to compare the differences in species diversity between pairs of samples. The software used and its version was QIIME (v1.80).

#### Principal coordinate analysis (PCoA)

QIIME (V1.80) software was used for PCoA to display the differences in community composition between three groups of samples. Samples with high similarity in community composition tend to cluster together, while samples with significant differences in community composition tend to separate.

The Linear Discriminant Analysis Effect Size (LEfSe) method was used to calculate and plot Linear Discriminant Analysis (LDA) scores to reflect the microbial communities significantly influencing the three groups of samples. Significant differences between species were considered when LDA ≥ 2.0.

PICRUSt2 (Phylogenetic Investigation of Communities by Reconstruction of Unobserved States) software was used to predict the functions of microbial communities. PICRUSt2 is a software program that predicts functional abundance based on marker gene sequences, including the KEGG, COG, MetaCyc metabolic pathways, and others. PICRUST2 is a software to predict functional abundance based on tagged gene sequences. The abundance prediction results of KEGG functions in bacterial communities were obtained by PICRUST2. Functions were named by KO ID, which represented specific functional genes, and then three levels of information of metabolic pathways were obtained based on the information in the KEGG database, and the abundance table of each level was obtained separately. After the predictions of functions were obtained for all samples, a Wilcoxon test (two groups of samples) was used to find the differential functions between the groups, which were presented graphically.

#### Statistical analysis

For two groups, a Wilcoxon test was used, and for more than two groups, a Kruskal–Wallis (KW) test was applied; both are nonparametric tests.

## Results

### Quality analysis of sequencing results and OTU analysis

**Figure 1:**
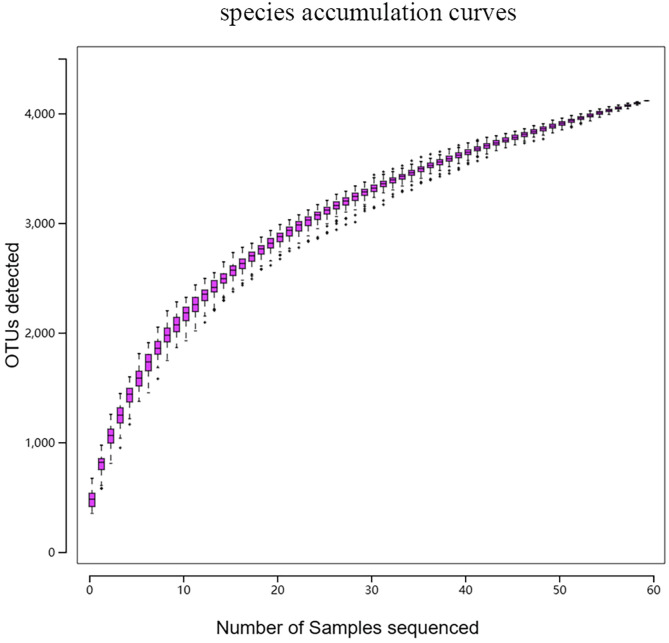
Species accumulation curves.

**Figure 2:**
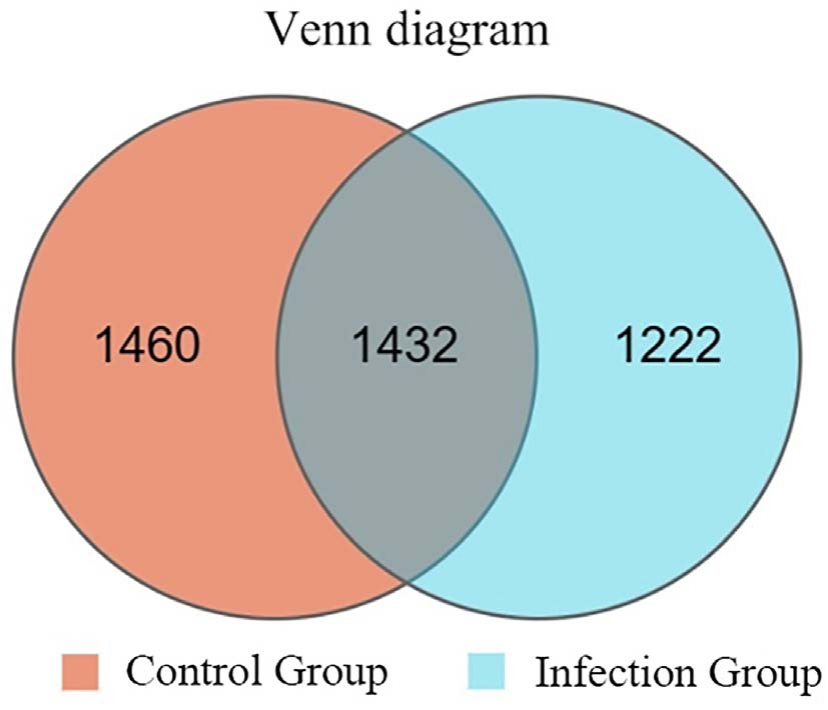
OTU Venn diagram.

**Figure 3:**
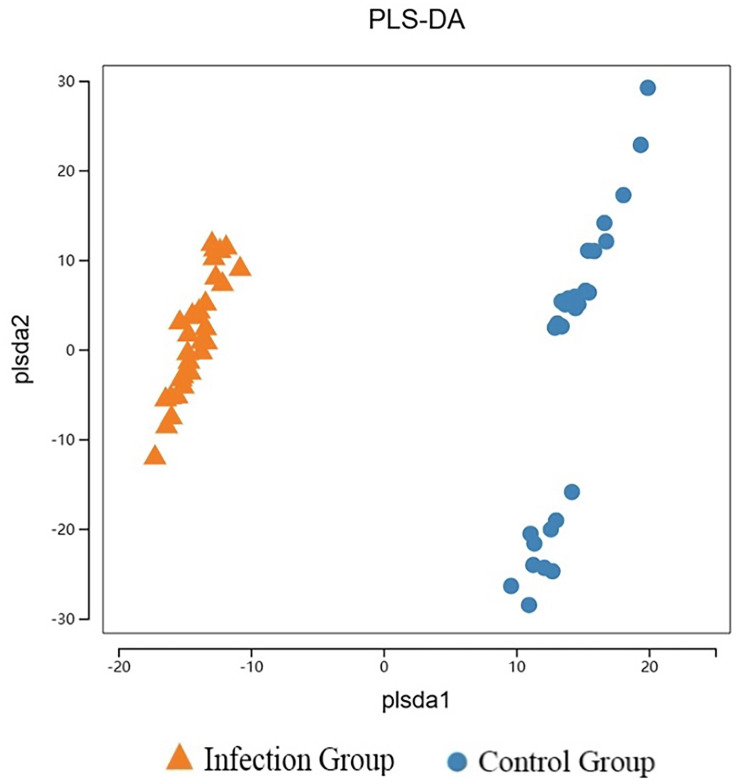
PLS-DA analysis diagram based on OTU abundance.

The species accumulation curve, as shown in Figure 1, gradually flattens out as the number of samples increases, indicating that the sequencing depth and coverage meet the requirements for subsequent analysis. From Figure 2, it can be observed that the number of observed OTUs in the experimental group is fewer than in the control group. The number of shared OTUs between the two groups of samples was 1,432, with 1,460 unique OTUs in the control group and 1,222 unique OTUs in the experimental group. Further, based on OTU abundance, Partial Least Squares Discriminant Analysis (PLS-DA) was performed to analyze the similarity of the sample compositions between the two groups. As shown in Figure 3, samples within the same group cluster closely, indicating strong similarity. However, there was a significant separation between the infected group and the control group, indicating a distinct change in the gut microbiota composition of lambs after infection with *Nematodirus oiratianus*.

### α-Diversity analysis

**Figure 4:**
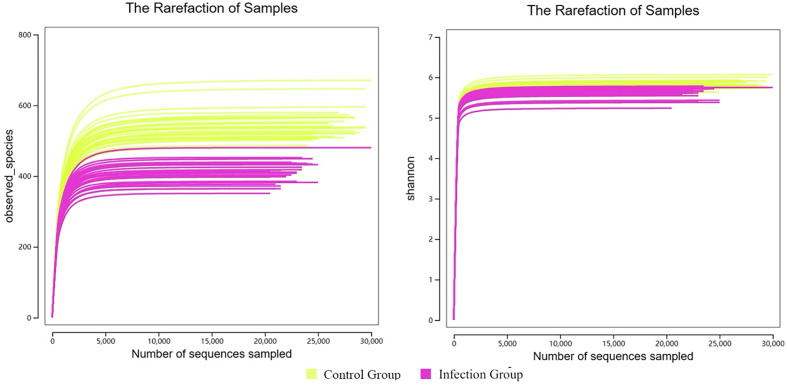
α-Diversity dilution curve.

**Figure 5:**
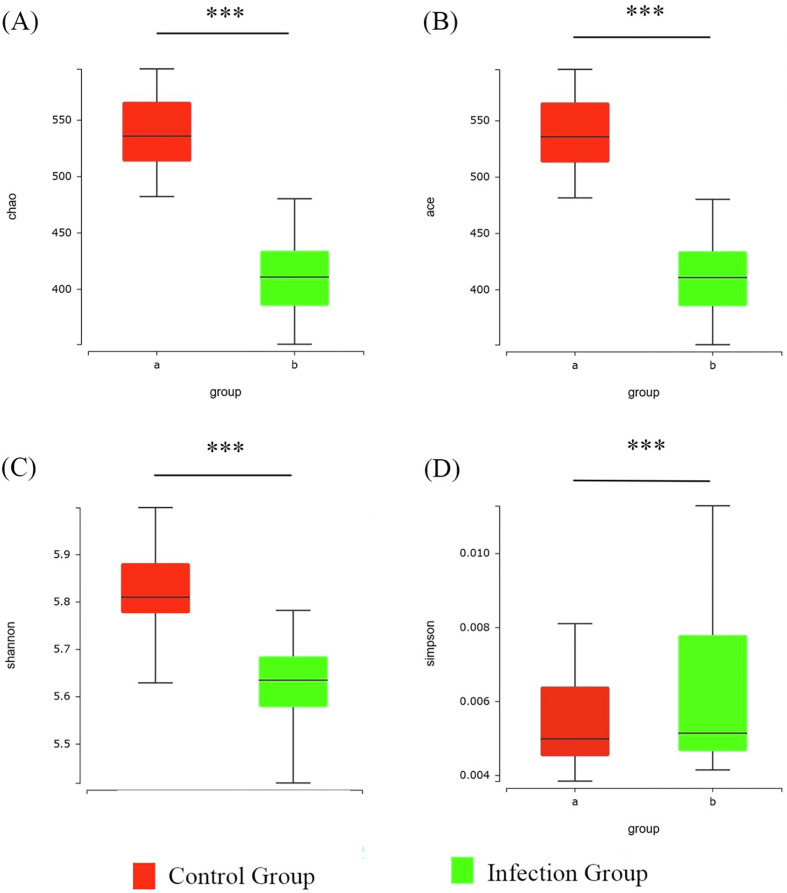
α-Diversity analysis. (A) Chao index; (B) ACE index; (C) Shannon index; (D) Simpson index; a, Control Group; b, Infection Group. ***p ≤ 0.001.

The results of the α-diversity analysis showed that sequencing depth and coverage can reflect the information of the vast majority of microbial species in the samples (Fig. 4). To further compare the differences in species richness between the infected group and the control group, the Chao, Ace, Shannon, and Simpson indices were used to measure the α-diversity of species. Figure 5 shows that, compared to the control group, the Chao, Ace, and Shannon indices of the infected group were significantly lower (*p* < 0.05), but the Simpson index of the infected group was slightly higher than that of the control group (*p* > 0.05). Combined with the characteristic of the Simpson index for measuring dominance, it is suggested that there may be a situation where a few taxonomic groups dominate in the infected samples. This result suggests that there is a significant change in the α-diversity of the gut microbiota in lambs after infection with *Nematodirus oiratianus*.

### β-Diversity analysis

**Figure 6:**
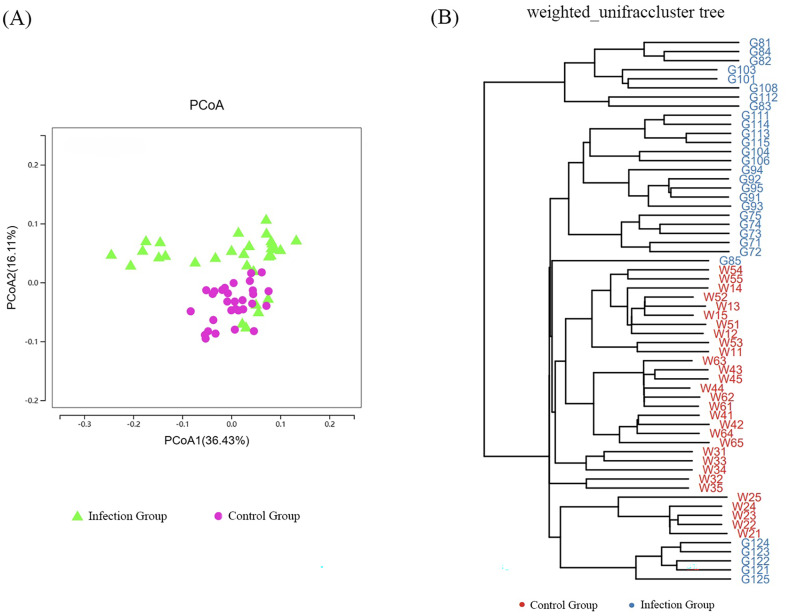
β-Diversity analysis. (A) Principal Coordinates Analysis (PCoA) according to the Unweighted UniFrac distance; (B) Clustering analysis according to the Unweighted pair-group method with arithmetic means.

The β-diversity of intestinal microbiota in the two groups of lambs was analyzed using PCoA based on Weighted UniFrac distances. As shown in Figure 6A, the contribution rates of the first and second principal components (PCoA1 and PCoA2) to the sample differences between the control group and the infected group were 36.43% and 16.11%, respectively. Further cluster analysis using the Unweighted Pair Group Method with Arithmetic Means (UPGMA) indicated that the control group and the infected group were generally located on different branches, with a few outliers. This result is consistent with the PCoA, demonstrating that there are significant differences in the intestinal microbiota structure between the lambs in the *Nematodirus oiratianus* infection group and those in the normal control group (Fig. 6B). These results indicate that infection with *Nematodirus oiratianus* can alter the β-diversity of the intestinal microbiota, resulting in significant microbial community differences among multiple samples.

### Comparison of intestinal microbiomes at different taxonomic levels

**Figure 7:**
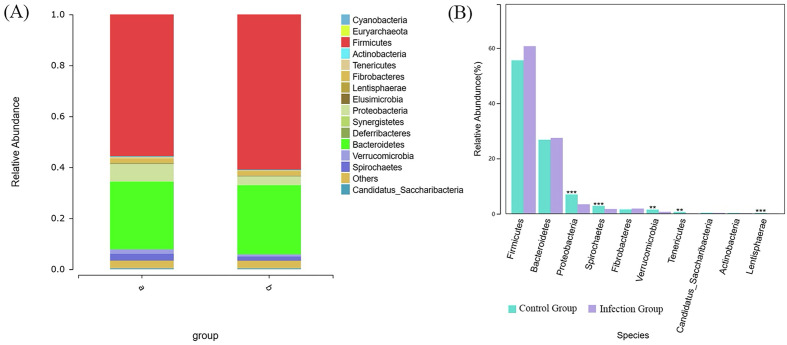
Analysis of intestinal flora composition at the phylum level. (A) Community structure of bacteria from different treatment groups in phylum level; (B) Comparison of key species differences; a, Control Group; b, Infection Group. ***, *p* ≤ 0.001; **, *p* ≤ 0.01; *, *p* ≤ 0.05.

The composition analysis of the intestinal microbiota at the phylum level in the two groups of lambs is shown in Figure 7A. The top five most abundant phyla were Firmicutes (55.56%), Bacteroidetes (26.79%), Proteobacteria (6.98%), Spirochaetes (2.83%), and Fibrobacteres (1.57%). These five phyla accounted approximately for 90% of the entire intestinal microbiota, representing the dominant bacterial groups in the gut. After infection with *Nematodirus oiratianus*, the proportions of Firmicutes (60.71%), Bacteroidetes (27.48%), and Fibrobacteres (1.88%) in the gut increased, although these changes were not statistically significant. However, the proportions of Proteobacteria (3.43%), Spirochaetes (1.72%), Lentisphaerae (0.11%), Verrucomicrobia (0.72%), and Tenericutes (Mycoplasmatota) (0.15%) decreased significantly, with these differences being statistically significant (*p* < 0.05) (Fig. 7B).

**Figure 8:**
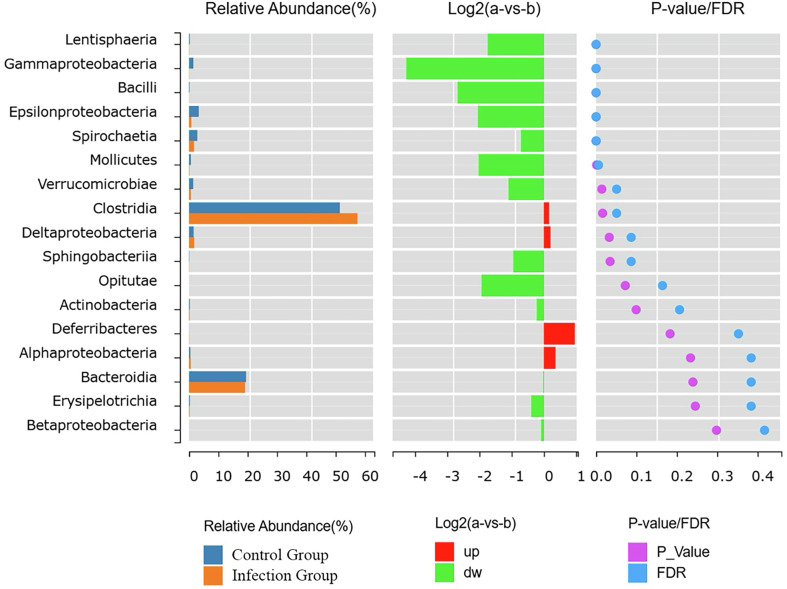
Analysis of species differences at the class level.

At the class level, compared to the control group, the infected group showed a significant decrease in the relative abundance of Lentisphaerae, Gammaproteobacteria, Bacilli, Epsilonproteobacteria, Spirochaetia, Mollicutes, and Verrucomicrobia (*p* < 0.05). In contrast, the relative abundance of Clostridia and Deltaproteobacteria significantly increased compared to the pre-infection levels (*p* < 0.05) (Fig. 8).

**Figure 9:**
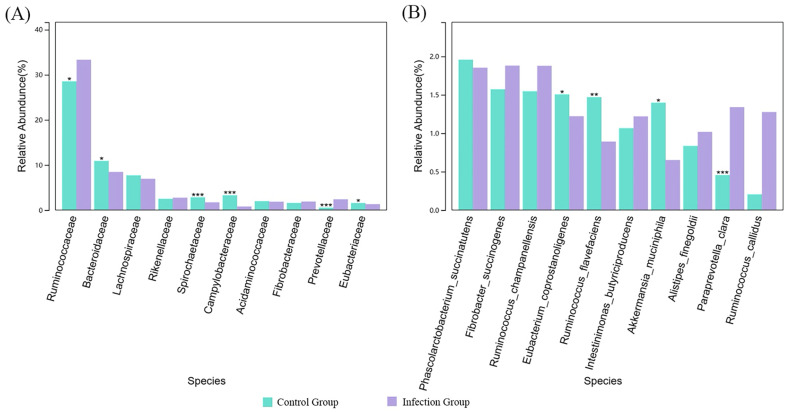
Comparison of key species differences. (A) Family level; (B) Species level. ***, *p* ≤ 0.001; **, *p* ≤ 0.01; *, *p* ≤ 0.05.

As shown in Figure 9A, at the family level, compared to the control group, the abundance of Prevotellaceae (*p* < 0.01) and Ruminococcaceae (*p* < 0.05) significantly increased in the intestines of lambs infected with *Nematodirus oiratianus*. In contrast, the abundance of Spirochaetaceae, Campylobacteraceae (*p* < 0.01), Bacteroidaceae, and Eubacteriaceae (*p* < 0.05) significantly decreased. Further analysis at the species level to identify key species (Fig. 9B) showed that, compared to the control group, the relative abundance of *Paraprevotella clara* (*p* < 0.01) significantly increased in the infected group. In contrast, the relative abundances of *Ruminococcus flavefaciens* (*p* < 0.01), *Eubacterium coprostanoligenes* (*p* < 0.05), and *Akkermansia muciniphila* (*p* < 0.05) significantly decreased.

### Analysis of differences in microbial composition among sample groups

**Figure 10:**
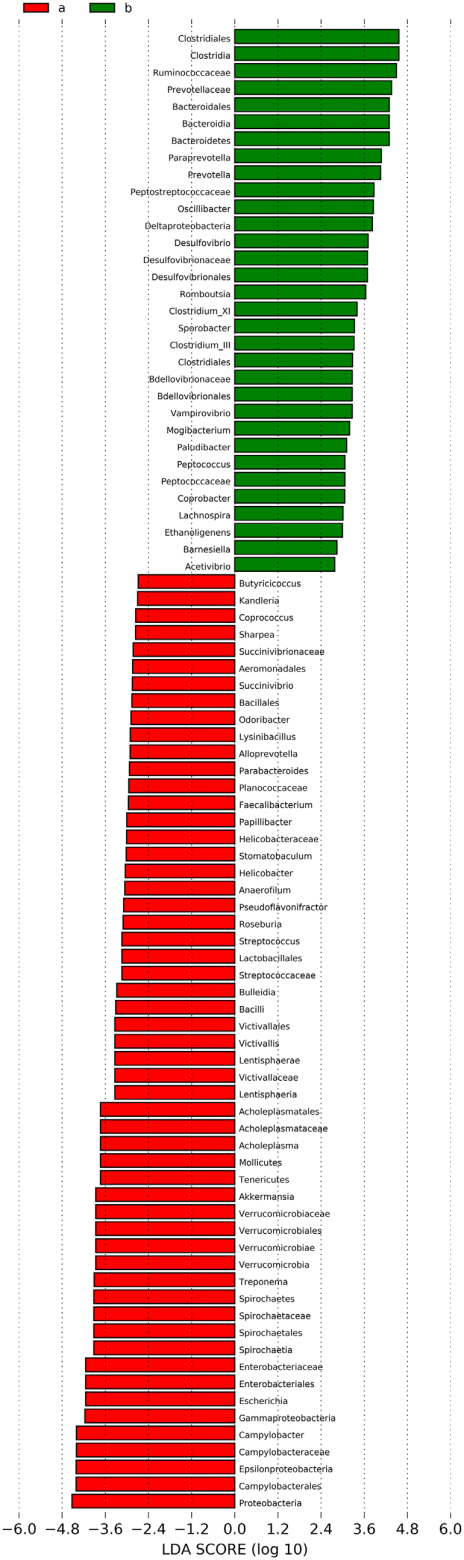
Linear discriminant analysis (LDA) effect size (LEfSe) analysis to screen specific dominant bacterial taxa of the two groups (a, Control Group; b, Infection Group). Distribution histogram based on LDA analysis (LDA score > 2 and *p* < 0.05). The abscissa represents the LDA score, and the ordinate represents the differential bacterial taxa.

To further analyze whether there are differences in specific dominant bacterial groups between the infected and control groups, LEfSe analysis and LDA (LDA > 2, *p* < 0.05) were performed to identify biomarkers. The results showed significant differences in intestinal microbiota between the two groups (Fig. 10). In the uninfected group, 55 taxa, including Proteobacteria, Bacteroides, and Campylobacterales, showed significant differences. In the infected group, 32 taxa showed significant differences, including Clostridiales, Firmicutes, Ruminococcaceae, and Clostridium cluster XI. Among these, Clostridiales exhibited the most significant differences.

### Assessment of predictive capability of microbial biomarkers

**Figure 11:**
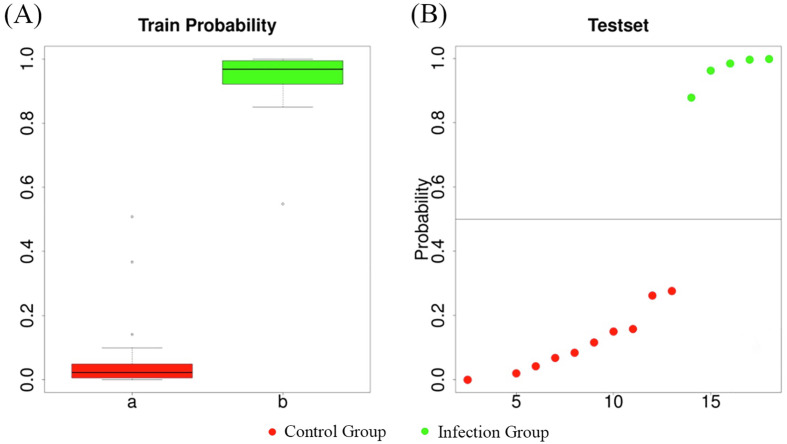
Randomized forest analysis. (A) Probability plot of the training set; (B) Distribution plot of the test set; a, Control Group; b, Infection Group.

To further evaluate the important differential taxa identified by the LEfSe analysis, the authors applied Random Forest to determine the reliability of the differential species in classifying the model based on probability values. According to the LEfSe results, a Random Forest analysis [8] was used to demonstrate the discriminative effect of combining four key differential taxa – *Desulfovibrio*, *Escherichia*, *Paraprevotella*, and *Campylobacter –* between the groups. As shown in Figure 11, the boxplots on the left side (Fig. 11A) indicate that the farther apart the boxes, the greater the difference between the groups. The test distribution plot on the right (Fig. 11B) shows that the two groups can be distinguished along the 0.5 line on the *y*-axis, indicating significant differences between the groups and a well-performing model.

**Figure 12:**
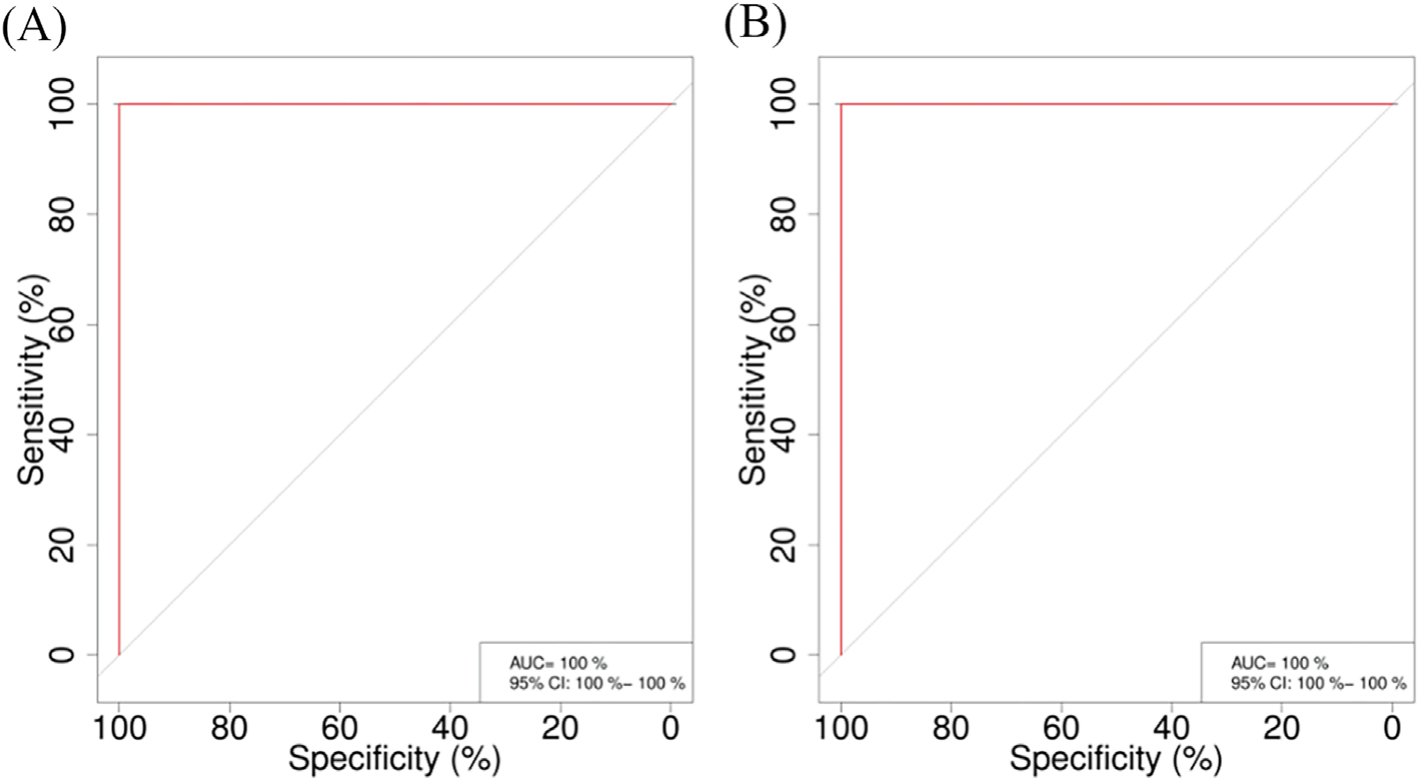
ROC curve analysis. (A) ROC of the training set; (B) ROC of the test set.

To determine the diagnostic value of the relative abundances of the differential genera identified by LEfSe and Random Forest analyses, ROC curve analysis [8] was performed on *Desulfovibrio*, *Escherichia*, *Paraprevotella*, and *Campylobacter*, and the area under the curve (AUC) was calculated. The results shown in Figure 12 indicate that the combined AUC of *Desulfovibrio*, *Escherichia*, *Paraprevotella*, and *Campylobacter* is 1, representing the optimal diagnostic indicator. This suggests that these bacterial genera have significant diagnostic value and may provide a theoretical basis for diagnosing whether lambs are infected with *Nematodirus oiratianus*.

### Prediction of microbial gene functions

**Figure 13:**
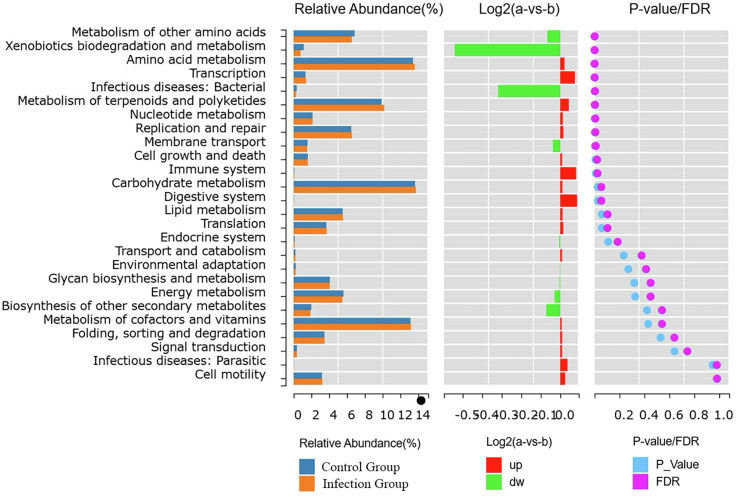
Analysis of functional differences.

To explore the differences in functional characteristics of intestinal microbial communities between the infected and control groups, we used the KEGG database to analyze the pathway abundance at Level 2 across six aspects: metabolism, genetic information processing, environmental information processing, cellular processes, organismal systems, and human diseases. Compared to the control group, the microbiota in the experimental group was primarily associated with metabolic pathways. Among these, 11 metabolic pathways, including other amino acid metabolism, xenobiotics biodegradation and metabolism, carbohydrate metabolism, and amino acid metabolism, showed significant differences between the two groups (*p* < 0.05). In addition, a decrease in the relative abundance of other amino acid metabolic pathways, biodegradation of xenobiotics and metabolic pathways, and an increase in the relative abundance of amino acid metabolic pathways, terpene and polyketide metabolic pathways, and nucleotide metabolic pathways were also found to be present after infection (Fig. 13).

## Discussion

Recently, the importance of the gut microbiome in host digestion, physiology, nutrition, and immunity has become increasingly prominent. An imbalance in the gut microbiome can lead to dysregulation of the immune system, damage to the intestinal mucosal barrier, and the development of chronic inflammatory diseases. Previous studies have found that the interaction between gut microbiota and parasites can alter the pathogenicity of both [30]. The intestinal environment in which they coexist is a key factor influencing the virulence of parasites. Simultaneously, parasitic infections can also affect the quantity and composition of the gut microbiota.

Moreover, relevant literature indicates that the mammalian gut can adapt to certain pathogens to some extent, including parasitic worms that are highly prevalent in many tropical countries [17]. However, as research on the gut microbiome deepens, we increasingly realize the immense challenge the immune system faces in distinguishing between commensal bacteria and pathogens. This challenge is particularly severe when there is a close association or competitive relationship between commensal bacteria and pathogens [10]. Thus, it is evident that pathogens may influence the host’s tolerance to normal microbiota and affect host regulation of these microbiotas, potentially leading to disease [24]. In our study, *Nematodirus oiratianus* infection significantly altered the abundance of intestinal microbiota in lambs, with the control group exhibiting a more diverse microbiota compared to the infected group. We hypothesize that this may be due to parasite invasion disrupting the host’s immune tolerance mechanisms towards the commensal gut microbiota, leading to an overreaction of the immune system and subsequent disruption of the normal microbiota.

According to existing research [11, 15, 41], parasitic infections are associated with reduced microbial diversity in the host gut. For example, in a study involving a canine animal model infected with *Toxocara canis* [31], the Ace, Chao, and Shannon indices showed that the richness, diversity, and evenness of the microbiota in the infected group were significantly lower than those in the control group. Holm and colleagues [13], through 16S sequencing analysis, found that chronic tapeworm infection in mice significantly reduced overall microbial diversity. However, this effect appeared to be reversible after the worms were cleared [14]. Another study assessing the changes in gut microbiota caused by fluke infection indicated that [16], despite the differences in the overall composition of the gut microbiota between the infected and control groups, there were no significant differences in species richness and diversity. Similarly, in this study, we found that the Ace, Chao, and Shannon indices all showed significant decreases following infection, indicating a notable change in species diversity. This reflects a temporary microbial dysbiosis within the host, likely due to the gut immune response triggered by the perception of parasitic invasion. This is consistent with previous research findings, where parasitic invasion of the host leads to a disruption of gut homeostasis, resulting in a range of disease symptoms.

In this study, we observed significant differences in the gut microbiota structure between the *Nematodirus oiratianus* infection group and the normal control group through PCoA and UPGMA analyses. Specifically, at the phylum level, the infected group had higher relative abundances of *Firmicutes* and *Bacteroidetes*, while the control group had a higher relative abundance of Proteobacteria. At the class level, the infected group exhibited a higher relative abundance of Clostridia. At the family level, the abundance of *Prevotellaceae* significantly increased, and the abundance of *Campylobacteraceae* significantly decreased in the infected group. At the species level, *Akkermansia muciniphila* had a higher relative abundance in the control group. Existing studies have shown that *Bacteroidetes* and *Firmicutes* can promote carbohydrate metabolism and energy absorption [22], and can also produce free radical scavengers and inflammatory regulators in the gut, thereby inhibiting oxidative stress and inflammatory responses. This effectively prevents the occurrence and progression of gastrointestinal diseases [25]. *Firmicutes* are an important component of probiotics and have beneficial properties for the host, such as promoting digestion, enhancing immunity, and inhibiting the growth of pathogenic bacteria [32]. At the same time, Rosa and colleagues’ [26] research suggests that the presence of roundworms is positively correlated with the abundance of *Firmicutes* bacteria. This is consistent with the higher expression of *Firmicutes* and *Bacteroidetes* observed in the infected group compared to the control group. Furthermore, studies have shown that *Bacteroidetes*, *Firmicutes*, *Actinobacteria*, and *Verrucomicrobia* can utilize carbohydrates from mucus as a carbon source [34]. Therefore, it can be inferred that following parasitic infection and the subsequent stimulation of mucus production, certain microbial groups may gain a competitive advantage. We hypothesize that the increase in Bacteroidetes observed in this study may be due to changes in mucus production following parasitic infection. *Proteobacteria* are considered characteristic of microbial dysbiosis. Shin *et al*. [29] reported that the abundance of *Proteobacteria* is low in the healthy human gut, but under certain pathogenic conditions, their proliferation is induced, leading to the production of pro-inflammatory factors and subsequent intestinal inflammation. In this study, the significant decrease in *Proteobacteria* may indicate a lower presence of pro-inflammatory factors and a slight imbalance in gut microbial homeostasis. There are reports that *Clostridia* and *Clostridiales* are microbial groups associated with host immunity [35, 36]. In this study, the significant changes in *Clostridia* suggest that *Nematodirus oiratianus* infection may affect the regulation of the host’s normal immune response. This could facilitate the repair of gut damage and the suppression of inflammation caused by *Nematodirus oiratianus* infection. In ruminants, *Prevotella* plays a key role in the degradation of ruminal proteins, particularly in the breakdown of oligopeptides, due to its rate-limiting dipeptidyl peptidase IV (DPP-IV) activity, which is responsible for oligopeptide cleavage [37]. The increase in the number of *Prevotella* in lambs infected with *Nematodirus oiratianus* may play an important role in host protein metabolism. *Akkermansia*, a Gram-negative bacterium, has recently been found to have a negative correlation with the incidence of appendicitis and inflammatory bowel disease. This may be related to certain anti-inflammatory factors, indicating a negative correlation with inflammatory responses [23, 33]. It is noteworthy that decreased levels of *Akkermansia muciniphila* have been observed in inflammatory bowel disease and metabolic disorders, suggesting its potential anti-inflammatory properties [5]. Evidence suggests that increased levels of *Akkermansia muciniphila* are associated with elevated levels of anti-inflammatory cytokines IL-10 and IL-4, as well as decreased levels of pro-inflammatory cytokines TNF-α and IFN-γ [3]. In addition to regulating immune function, *Akkermansia muciniphila* promotes mucus secretion [21], enhances mucosal barrier integrity, and strengthens barrier mechanisms, thereby contributing to overall gut health [7, 9]. We hypothesize that the low concentration of *Akkermansia muciniphila* observed in this study may indicate a thinner mucus layer, leading to weakened gut barrier function.

To analyze the functional differences between different groups, we used PICRUSt2 to predict microbial community functions. The functional difference analysis results showed that the microbiota were primarily associated with metabolic pathways. Previously, it was suggested that roundworm infection is related to changes in the metabolic potential of the pig gut microbiome [39]. In sheep [1], related unique bacterial genera (such as *Campylobacter*, *Bibersteinia*, *Mailhella*, and *Frisingicoccus*) were also found in the intestine after infection with whipworms, which also showed metabolic duality. Houlden’s [14] research showed that mice with chronic tapeworm infection exhibited changes in carbohydrate and amino acid metabolism, as well as a reduced capacity for nutrient absorption. Our research results similarly reflect these metabolic changes. In summary, these findings suggest that worm-mediated changes in the microbiome often lead to a reduced metabolic capacity of the gut, ultimately resulting in nutritional deficiencies.

On the other hand, based on the LEfSe results, we used Random Forest analysis to identify four key differential taxa. We found that combining *Desulfovibrio*, *Escherichia*, *Paraprevotella*, and *Campylobacter* resulted in an AUC of 1, indicating the optimal diagnostic indicator. These taxa can serve as reliable biomarkers to accurately predict worm infection in lambs.

Currently, some researchers have found that changes in the gut microbiota structure are not related to worm burden [40]. This suggests that there is a relatively persistent change in the gut microbiota during the initial stages of worm infection, and these changes remain even after the host reduces the worm burden. This finding is significant because most studies focus on changes in the gut microbiota during the current stage of worm infection, often overlooking the possibility that the host might have previously encountered and cleared the worm. Based on this hypothesis, future research must explore this aspect in greater depth. Building on our preliminary work, this project will aim to collaborate with other research institutions to obtain more accurate samples. This will lay the foundation for further in-depth studies on the impact of *Nematodirus oiratianus* on normal microbiota structure and the immune regulatory mechanisms of *Nematodirus oiratianus.*

## Conclusions

This study showed that the abundance and species diversity of the intestinal flora of lambs infected with *Nematodirus oiratianus* changed significantly. We also found significant differences in the structural composition of their intestinal flora before and after infection. KEGG analysis of the infected and control groups revealed that the flora was mainly associated with metabolic pathways. Finally, four diagnostic species were screened, which can be a good predictor of helminth infection. This study will lay a theoretical foundation for further research on how *Nematodirus oiratianus* infection affects the function of intestinal flora.
